# A Novel Untethering and Duraplasty Technique for Postsurgical Tethered Spinal Cord

**DOI:** 10.7759/cureus.34137

**Published:** 2023-01-24

**Authors:** Justin D Cohen, Robert A Ravinsky, Monis A Khan, Jake Sossamon, Terrence Kim, Kamal Woods, Robert Naruse, Ulrich Batzdorf, J. Patrick Johnson

**Affiliations:** 1 Department of Neurosurgery, Cedars-Sinai Medical Center, Los Angeles, USA; 2 Department of Orthopedics and Physical Medicine, Medical University of South Carolina, Charleston, USA; 3 Department of Neurologic and Orthopedic Surgery, University of Arizona College of Medicine - Phoenix, Phoenix, USA; 4 Department of Orthopedic Surgery, Cedars-Sinai Medical Center, Los Angeles, USA; 5 Department of Neurosurgery, Vetrae Inc., Dayton, USA; 6 Department of Anesthesiology, Cedars-Sinai Medical Center, Los Angeles, USA; 7 Department of Neurosurgery, University of California Los Angeles David Geffen School of Medicine, Los Angeles, USA

**Keywords:** spinal cord injury, intramedullary spinal cord tumor, progressive postsurgical myelopathy, duraplasty, tethered spinal cord

## Abstract

Progressive post-traumatic postsurgical myelopathy (PPPM) is a known entity that can occur months to years after the initial insult. Symptomatic patients can become myelopathic and have rapid and progressive neurological decline. Surgical correction of PPPM usually involves intradural exploration and lysis of adhesions that carries the risk of further injury to the spinal cord. In this manuscript, we provide a report of a patient presenting more than 50 years after the initial resection of an intramedullary tumor. Additionally, we present and describe a novel surgical technique for managing this difficult problem and restoring normal CSF dynamics.

## Introduction

Progressive post-traumatic postsurgical myelopathy (PPPM) following spinal cord injury, or iatrogenic injury, are well-known entities that can occur months to years after the initial insult. In 1976, Hoffman et al. described Post-Surgical Tethered Cord Syndrome (PS-TCS) that occurs when abnormal attachments within the thecal sac result in non-physiologic stretching of the neural elements [1]. This, in turn, may result in myelopathy and neurological deterioration.

PPPM has been well-described in the setting of intramedullary spinal cord tumor resection and secondarily occurs when the dorsal pial surface of the surgically exposed spinal cord adheres to the dorsal dura [2-6]. Surgical correction of PPPM usually involves intradural exploration and lysis of adhesions that carries the risk of injuring the spinal cord. In a review of the literature, a small case series by Smith et al. describes post-surgical tethering occurring 1-31 years after initial surgery [7]. To the best of our knowledge, there are no published English language reports of cervical spinal cord tethering discovered 50 years after intradural surgical intervention. 

In this article, we present a rare case of PPPM, presenting more than 50 years after the patient underwent resection of an intramedullary tumor of the cervical spine. Additionally, we present and describe a novel surgical technique for managing this difficult problem and restoring normal CSF dynamics. First, the adherent “dural placode” is resected to its adherent base and left attached to the spinal cord to create an island of dorsal dura and second, a generous and “billowing duraplasty" is created and the CSF cistern insufflated with saline prior to dural closure.

## Case presentation

**Figure 1:**
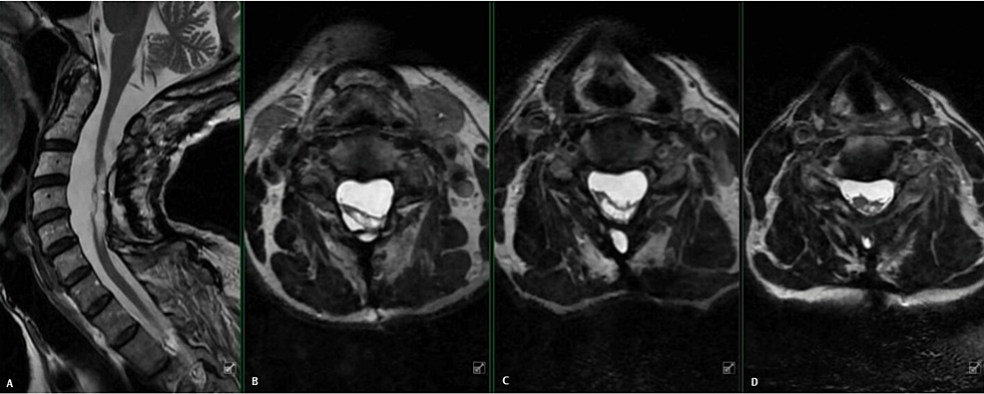
Preoperative MRI of the cervical spine with mid-sagittal view (A) and selected axial T2-weighted images at C3/4 (B), C4/5 (C) and C5/6 (D), demonstrating dorsal tethering of the cervical spinal cord to the overlying dura from approximately C3 to C7. There is eccentricity of the tethering to the left side, as well as a large central syrinx with marked atrophy of the cord.

**Figure 2:**
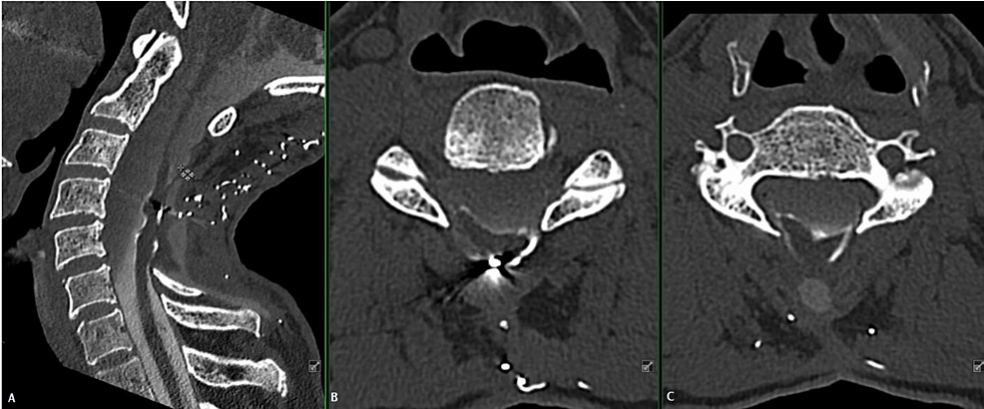
CT myelogram of the cervical spine with mid-sagittal view demonstrating a posterior laminectomy from C2 to C7 (A) and selected axial images at C3/C4 (B), and C4/C5 (C) with multiple areas of dorsal tethering of the spinal cord, as well as obstruction of CSF flow ventral to the cord, suggestive of an arachnoid cyst. Scatter is occurring due to numerous retained vascular clips from previous surgical interventions.

The patient was a 69-year-old male who presented with a history of slow, progressive, and profound left-sided weakness in the upper and lower extremities over the course of more than 20 years. The patient had undergone a posterior cervical laminectomy and intramedullary tumor resection 58 years earlier at 11 years old. followed by two subsequent intradural procedures at the ages of 18- and 32-year-old for detethering of the spinal cord. At the time of the presentation, he continued to work as an executive, retaining some left-sided hand grip strength. His past medical history was otherwise unremarkable. On physical examination, the patient demonstrated severe atrophy of the left upper extremity musculature including the reduced size of the biceps brachii, brachialis, coracobrachialis, and triceps brachii. Gait was grossly ataxic, and he favored weight bearing through the right lower extremity. His left handgrip strength was 2/5 but otherwise demonstrated 0/5 power in the remaining muscle groups of the left arm. He also demonstrated severe paresis of his left lower extremity. Power was uniformly 4/5 in all muscle groups of the right-sided extremities. Magnetic resonance imaging (Figures 1A-1D) and CT myelogram (Figures 2A-2C) of the cervical spine demonstrated a severely distorted and atrophic spinal cord from the C4 to C6 levels. The cord was dorsally adherent to the overlying dura, eccentric to the left side, while the right side appeared less affected.

After informed consent was obtained, the patient was taken to the operating room and underwent general anesthesia with fiberoptic endotracheal intubation. Ancef and Decadron were administered prior to skin incision. Intraoperative monitoring with somatosensory evoked potentials (SSEPs) and motor evoked potentials (MEPs) were performed, with baseline measurements recorded prior to positioning. The baselines were asymmetric in concordance with the clinical exam. The patient was positioned prone with his head immobilized in three-point skull fixation. He was prepped and draped in the usual sterile fashion. The previous midline incision was reopened and the prior laminectomy site from C3-5 was identified. A subfascial fluid collection was encountered and a small defect in the dorsal dura was identified within the laminectomy defect, consistent with a pseudomeningocele. The laminectomy was extended cranially and caudally to expose C2 to C6 and virgin dura.

**Figure 3:**
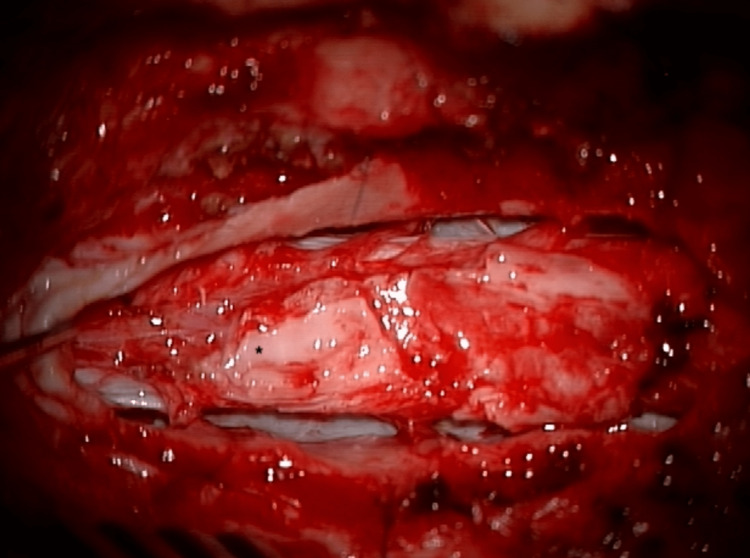
Intraoperative photograph of dural placode * dural placode

The dura was opened cephalad and caudal to the location of tethering, as noted on preoperative imaging, and dural tack-up sutures were placed to expose the underlying spinal cord with a segment of adherent dorsal dura. The free dorsal edge of the adherent dura was identified and gently cut away from the underlying spinal cord leaving a central area consisting of a “dural placode” that could not be safely removed from the spinal cord (Figure 3). The dura was circumferentially released around the adherent dural placode until the spinal cord was completely released. The dentate ligaments were also sectioned, and the spinal cord was inspected for any arachnoidal adhesions that were circumferentially dissected from the cord. A large arachnoid cyst ventral to the spinal cord was also fenestrated in multiple places which allowed the spinal cord to shift ventrally from its dorsal tethering. The resultant untethering of the entirety of the spinal cord allowed the spinal cord to shift into the ventral aspect of the spinal canal.

**Figure 4:**
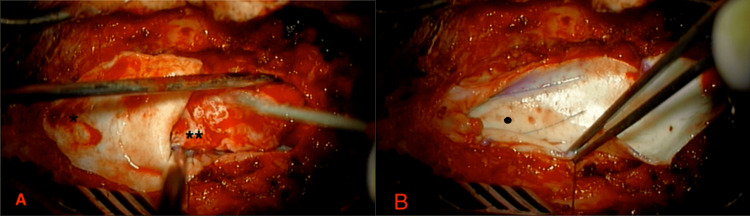
(A) Intraoperative photograph of the “Billowing Duraplasty” being sutured in place. (B) The completed sutured duraplasty. * represents allograft ** represents the underlying adherent placode Black dot shows completed sutured duraplasty

A large and billowing duraplasty with AlloDerm allograft (human tissue alloderm, LifeCell) was placed over the dural defect and sutured securely into position with a running 5-0 Prolene suture (Figures 4A, 4B). Prior to completing the duraplasty, the intradural space was insufflated with warm saline to create a “billowing” effect. Once the dura was closed, a Valsalva was performed, and no CSF leak was observed. Fibrin glue was applied along the suture line in order to reinforce the repair and create a water-tight seal. The wound was then copiously irrigated with antibiotic solution and vancomycin powder was applied. The wound was closed in anatomic layers with a myofascial advancement flap from the scarred posterior paraspinal region. A dorsal epifascial drain was left in place. The skin was closed with Steri-Strips and sterile dressings were applied.

At the end of the procedure, the patient was extubated and transferred to the post-anesthesia care unit in stable condition. The SSEPs and MEPs remained stable throughout the case.

**Figure 5:**
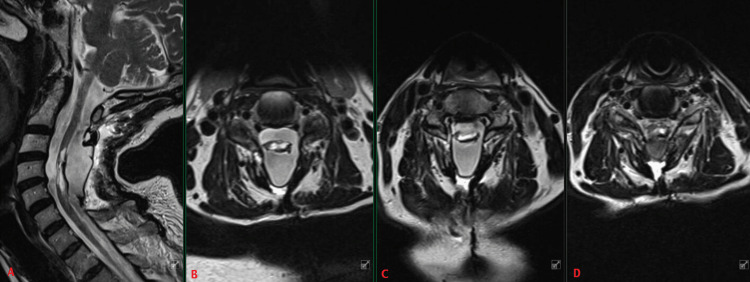
Postoperative MRI of the cervical spine with mid-sagittal (A) and selected axial T2-weighted images at C3/4 (B), C4/5 (C) and C5/6 (D) demonstrating a more ventral position of the cord compared to the preoperative imaging studies, and restoration of space and CSF fluid volume within the dorsal cistern. There is increased space between the spinal cord and the reconstructed dura, which has been facilitated by the “billowing duraplasty.” Note the presence of the retained, adherent dural placode on the dorsal aspect of the spinal cord, represented by the region of low signal on the T2-weighted images, best seen on the mid-sagittal slice.

Postoperatively, the patient was admitted to the surgical ward at our institution. His objective neurologic and ambulatory status was unchanged compared to his preoperative exam; however, he subjectively noted that he felt some improvement in his left upper extremity paresthesia. A post-surgical MRI study was performed to evaluate the quality of the detethering and evaluate the degree of restoration of the dorsal CSF cistern (Figures 5A-5D). He was discharged to a spinal cord rehabilitation facility on post-operative day 3 and spent 10-days at the rehab center. At time of manuscript, the patient is 1.5 years post-surgery and is doing well. The patient reports no further progressive decline in neurological function.

## Discussion

The syndrome of PPPM following spinal cord injury is an increasingly recognized entity, with a prevalence of 0.3% to 3.2% [8]. Lee et al. reviewed 40 patients with PPPM and found that trauma was the most common cause, but five patients were included in their series who had developed PPPM after previous tumor resection [8]. The time interval between the index surgical procedure and symptom onset may be highly variable in this population and data suggests that the development of clinically significant spinal cord adhesions or symptoms may take years or even decades to result. Raco et al. reviewed 202 patients that underwent removal of an intramedullary tumor from 1972 to 2003, and found cord tethering complicated 17% of the cases, in which 5% required reoperation [9]. However, this study did not elucidate the exact time frame from surgical intervention to the development of cord tethering. Smith et al. reported a smaller occurrence of five patients who developed cervical tethering 1-31 years (average 11 years) after the initial intradural intramedullary procedures [7,10,11].

Most of the literature on tethered cord syndrome reports on the lumbosacral spine in patients with primary myelodysplasias. It has been proposed that the time course and severity of symptoms are likely related to the degree of traction and tethering of the neural elements [12]. Smith et al. postulated that repeated movements of the head and neck with a relatively fixed spinal cord resulted in the tension of delicate neural structures and resulted in delayed/progressive cord ischemia with variable onset of symptoms [7]. It is possible that the risk of downstream intradural postsurgical tethering may be reduced by limiting the degree of surgical trauma to the intradural space at the time of index surgery and achieving a close approximation of the meningeal layers in order to decrease post-surgical scarring and formation of adhesions. It is known from spinal cord injury research that after any traumatic insult, the spinal cord undergoes reactive gliosis, extracellular matrix deposition, and scar formation that may further contribute to the tethering of the cord and result in injury to the spinal cord [13].

The goal of surgical correction of PPPM is to free the spinal cord circumferentially from any adhesions. It is common practice to perform microdissection of adherent scar tissue off of the spinal cord; however, this technique can be dangerous if there is not a clear plane between the spinal cord and the pia-arachnoid. An alternative method is described in this manuscript and includes leaving the adherent dura attached to the spinal cord and creating a “dural placode.” This technique reduces spinal cord manipulation, maximizes detethering, and can be safely used in treating PPPM.

It is unknown if retaining a placode of dura adhering to the dorsal aspect of the spinal cord may represent a potential locus for future tethering. To the best of our knowledge, there is no literature to either support or refute this possibility. However, the intended purpose of creating a “billowing duraplasty” is to expand the CSF cistern and create physical distance between the dorsal aspect of the cord with its adhering placode and the allodermaterial used to perform the duraplasty. It is our hypothesis that the physically enlarged dorsal CSF cistern will be protective with respect to future retethering.

While there exists a limited amount of literature from which conclusions may be drawn, based on the experience of the senior authors, we believe that there are a number of risk factors that may predispose individuals to develop PPPM (Table 1). Surgical risk factors include over-drainage of cerebrospinal fluid, aggressive manipulation/dissection of the spinal cord, improper dural closure technique, and failure to restore the arachnoid and/or pial membrane. Agarwal et al. reported a case of Tethered Cord Syndrome (TCS) associated with chronic over-drainage of CSF in a patient with a ventriculoperitoneal shunt and hypothesizes that a “dry” CSF space increases the chance of tethering [10].

**Table 1:** Proposed risk factors for the development of progressive posttraumatic postsurgical myelopathy (PPPM)

Risk Factors
· Dry CSF cisterns
· Close proximity of spinal cord and dura
· Failure to restore the integrity of the arachnoid membrane
· Increased extent of intradural surgical insult
· Multiple revision procedures
· Presence of concomitant structural anomalies
· Spinal cord injury
· Suture material and synthetic dura chosen

Prior studies have extrapolated different closure techniques and their influence on the development of PPPM. Goto et al. examined two different dural closure techniques, comparing two patient groups in the context of ependymoma resection, and found evidence that closure technique and surgical material utilized affected rates of postoperative tethering at a statistically significant level. Specifically, they found that within the study period, a technique of pial suturing, dural closure with Gore-Tex assisted patch grafting and expansile laminoplasty demonstrated 0% incidence of adhesive dural tethering or neurologic deterioration. In comparison, the cohort of patients that had undergone direct layer-to-layer closure of the three meninges and laminoplasty demonstrated a 47% rate of postoperative dural adhesion, and 29% experienced postoperative neurologic deterioration [14]. Additionally, in an animal study by Park and Tator, rats in a spinal cord injury model that had undergone decompression and duraplasty demonstrated reduced postoperative spinal cord tethering when Gore-Tex was used as duraplasty material when compared to other materials [15].

Given the proposed risk factors for PPPM described above, we recommend that the following principles be considered, and technical measures taken during the course of treating a patient with PPPM. Surgical intervention is indicated in a neurologically deteriorating patient, whose neurologic demise can be explained by tethering of the spinal cord. The objectives of surgery include complete detethering of the spinal cord in order to relieve traction/vascular insult, restoration of normal CSF flow, and correction of any concomitant intradural pathology, such as the presence of compressive arachnoidal cysts. Prevention of recurrent tethering is potentially accomplished at the time of surgery by leaving a dural placode as necessary, and use of the “billowing duraplasty” technique in order to create physical space between the dorsal spinal cord and the dura/duraplasty material.

## Conclusions

PS-TCS is a rare, but serious condition that carries significant morbidity and is difficult to treat. The presentation can be months to decades after the initial surgery. Major risk factors associated with PS-TCS include CSF over drainage, the extent of intradural surgical insult, and choice of suture material/synthetic dura. Surgical intervention is indicated in patients with progressive neurological or functional decline. Surgical untethering is best achieved, in high-risk cases like the one presented here, by leaving the adherent “dural placode” as an island along the spinal cord and creating a large “billowing duraplasty” to increase the CSF space between spinal cord and dura.
